# Changes in Hepatitis A seroprevalence over 11 years and the impact of the National Vaccination Program in Bursa, Türkiye

**DOI:** 10.1007/s11845-026-04346-7

**Published:** 2026-03-31

**Authors:** Büşra Çalışır, Ayşe Melda Payaslıoğlu, İmran Sağlık

**Affiliations:** Department of Medical Microbiology, Bursa Uludağ University, Bursa, Turkey

**Keywords:** Hepatitis A, Seroprevalence, Vaccination, Immunization programs

## Abstract

**Background:**

Hepatitis A virus (HAV) infection remains a significant cause of acute viral hepatitis worldwide. Türkiye has transitioned from historically high endemicity to an intermediate-endemic setting, and the national childhood vaccination program introduced in 2012 has further influenced epidemiological patterns.

**Methods:**

We retrospectively analyzed 28,067 patients tested for anti-HAV IgG and IgM at Bursa Uludağ University Hospital between 2014 and 2024, a large tertiary-care referral center. Serological assays were performed using chemiluminescent microparticle immunoassays. Results were categorized by age, sex, and year of testing. IgM-positive cases were further evaluated for clinical and laboratory correlation.

**Results:**

The mean age was 36.1 years, and 51.7% were male. Overall, anti-HAV IgG seropositivity was 68.97%, ranging from 31.82% in the 21–25-year age group to 99.66% among those ≥ 60 years. Children born in 2012 or later (vaccine-eligible cohort) exhibited an anti-HAV IgG seropositivity rate of 93.3%, with no anti-HAV IgM positivity. Anti-HAV IgM was detected in 0.26% (72/28,067); however, only 19 cases (26.4%) fulfilled the criteria for acute hepatitis A, and 42% occurred in August–September. Remaining IgM reactivity was mostly asymptomatic/unlikely acute HAV or possible false positivity, frequently accompanied by HBV/HCV infection, EBV infection, autoimmune disease, or malignancy. Cut-off indices were significantly higher in confirmed acute cases (*p* < 0.001).

**Conclusions:**

Bursa, Türkiye, continues to exhibit intermediate HAV endemicity. The childhood vaccination program supports its effectiveness by demonstrating high seropositivity and the absence of IgM positivity in the vaccine-eligible cohort; however, false-positive IgM reactivity underscores the importance of clinical and laboratory confirmation.

## Introduction

Hepatitis A virus (HAV) infection is an acute, typically self-limiting liver disease caused by a single-stranded RNA virus of the Picornaviridae family [1, 2]. Transmission occurs mainly via the fecal–oral route, most commonly through ingestion of contaminated food or water or via close person-to-person contact [1–3]. The clinical course varies by age: infection in early childhood is often asymptomatic, whereas adolescents and adults more frequently develop overt clinical disease, and severity tends to increase with age. Although rare, fulminant hepatitis and fatal outcomes can occur [1, 3, 4]. Approximately 70% of infections in children under 6 years are asymptomatic and jaundice is uncommon in this age group, while more than 70% of older children and adults develop jaundice during acute HAV infection [2, 3].

Recent Global Burden of Disease (GBD) estimates indicate that acute hepatitis A remains highly prevalent worldwide, with approximately 160.86 million incident cases in 2021. Earlier GBD 2019 estimates cited by the World Health Organization (WHO) reported ~ 159 million acute HAV infections and ~ 39,000 deaths associated with HAV globally in 2019 [5, 6]. The prevalence of HAV infection is strongly influenced by socioeconomic conditions, access to safe drinking water, and sanitation and hygiene infrastructure [2, 6, 7]. While improvements in sanitation, food safety, and vaccination coverage have contributed to a global decline in incidence, HAV still poses a substantial public health burden, particularly in low- and middle-income countries [2, 6].

The endemicity level of hepatitis A within a population is determined by age-specific anti-HAV IgG seroprevalence. WHO classifies regions into high, intermediate, low, or very low endemicity using age-based seroprevalence thresholds [6]. In line with WHO recommendations, universal childhood vaccination is advised in settings of intermediate endemicity, whereas in low and very low endemicity settings vaccination is generally prioritized for high-risk groups and susceptible individuals [6].

Türkiye has been classified as an intermediate endemic country in global reports [6, 8]. The hepatitis A vaccine was incorporated into the national childhood immunization Schedule in 2012 and has since been administered in two doses at 18 and 24 months of age [9]. Reliable epidemiological data are essential for guiding vaccination strategies, and age-specific seroprevalence rates, in particular, provide robust indicators of the current epidemiological status of hepatitis A within a country [10].

The present study aimed to evaluate the overall and age-specific seroprevalence of HAV over an 11-year period in Bursa, the fourth largest city in Turkey, using data from a tertiary university hospital. In addition, the study assessed the impact of the national routine vaccination program on HAV seroprevalence.

## Materials and methods

This retrospective study included a total of 28,067 patients who presented to Bursa Uludağ University Faculty of Medicine Hospital between January 1, 2014 and December 31, 2024 and were tested for anti-HAV IgM and anti-HAV IgG antibodies in the Department of Medical Microbiology Laboratory for various indications. For patients with more than one serum sample, only the results of the first test were included in the analysis.

Serological testing was performed using the ARCHITECT HAVAb IgM and HAVAb IgG chemiluminescent microparticle immunoassays (CLIA) on the Architect i2000SR platform (Abbott Diagnostics, Abbott Park, IL, USA). According to the manufacturer’s instructions, results were interpreted as positive at values of ≥ 1.00 signal-to-cutoff index (S/CO) for anti-HAV IgG and > 1.21 S/CO for anti-HAV IgM.

Demographic characteristics (date of birth, age, and sex) of the patients were retrieved from the hospital information system and patient records. For those with reactive anti-HAV IgM results, clinical features (including jaundice, dark urine, pale stool, fever, abdominal pain, nausea, vomiting, malaise, fatigue, and anorexia), antibody levels, and liver function tests (ALT, AST, and bilirubin) were evaluated. Cases with reactive anti-HAV IgM results but without a clinically compatible acute hepatitis presentation and without supportive biochemical evidence (e.g., elevated ALT and/or total bilirubin) were considered unlikely to represent acute hepatitis A (possible non-acute/persistent IgM or false-positive reactivity) and were evaluated separately, in line with previous recommendations [11, 12]. Information regarding individual vaccination status was not accessible.

This study was approved by the Bursa Uludağ University Clinical Research Ethics Committee (Decision No: 2025/6–29).

All data were analyzed using IBM SPSS Statistics, Version 24 (SPSS Inc., Chicago, IL, USA). Descriptive variables were summarized as frequencies and percentages. Continuous variables were expressed as mean ± standard deviation (SD) with range values. Comparisons of categorical variables were primarily performed using Pearson’s chi-square (χ²) test. For contingency tables with expected cell counts < 5 (e.g., acute hepatitis A by age groups), Fisher’s exact test was applied. In addition, a chi-square test for trend was applied to evaluate age- and year-related changes in IgG seropositivity. A *p*-value < 0.05 was considered statistically significant.

## Results

### Study population and overall serology

A total of 28,067 patients were tested for hepatitis A serology between 2014 and 2024. The mean age was 36.1 ± 21.1 years (range 0–96), and 51.7% were male. Anti-HAV IgG seropositivity was detected in 68.9% of patients (19,357/28,067) and was significantly higher in males than in females (70.5% vs. 67.4%, *p* < 0.001).

Anti-HAV IgM positivity was identified in 72 individuals (0.26%). Clinically confirmed acute hepatitis A (based on compatible clinical features and supportive laboratory findings) was documented in 19 patients, corresponding to 26.4% of IgM-positive cases and 0.07% of the overall study population.

### Age-specific distribution

**Fig. 1:**
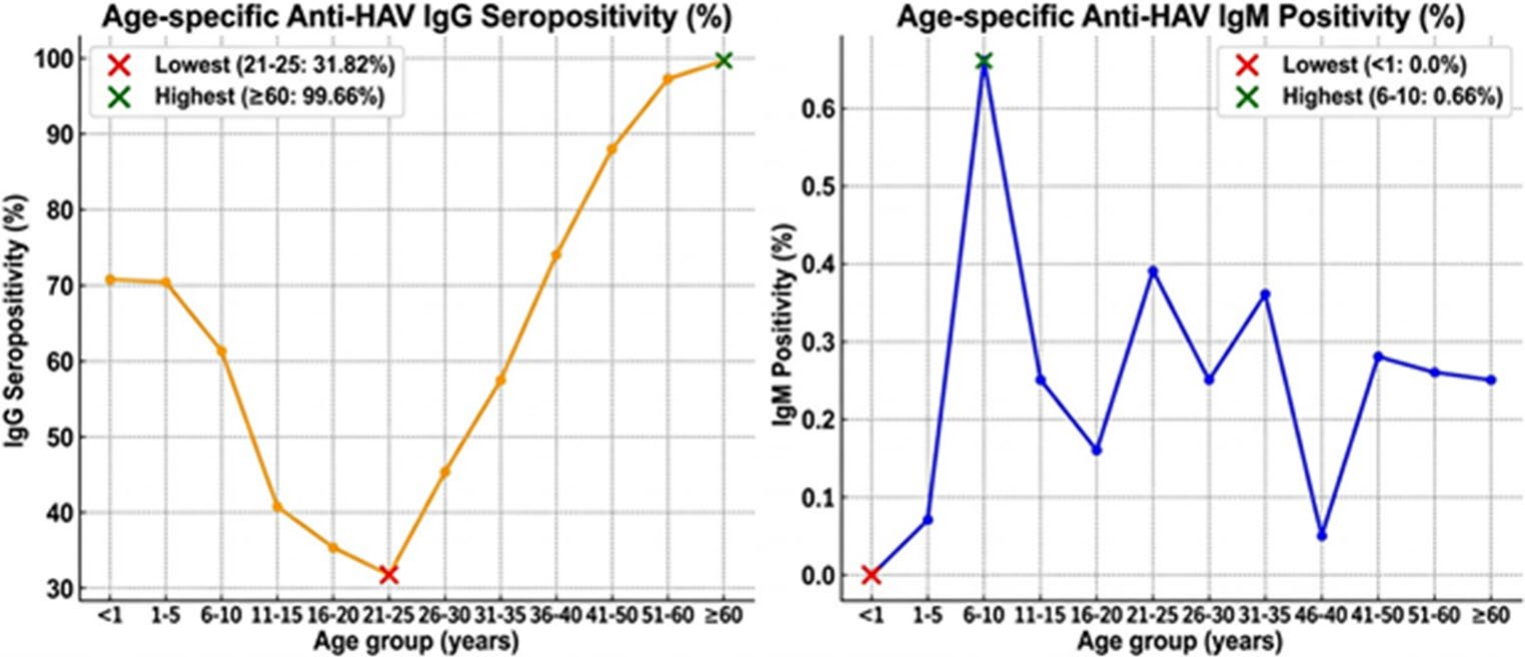
Age-specific anti-HAV IgG and IgM positivity rates (2014–2024). Anti-HAV IgG seropositivity showed a pronounced age-related pattern, with the lowest prevalence observed in the 21–25-year age group (31.82%), followed by a steady increase with advancing age. IgG positivity exceeded 70% by ages 36–40 and remained consistently high (≥88%) among individuals aged ≥41 years. In contrast, anti-HAV IgM positivity remained low across all age groups, peaking in children aged 6–10 years (0.66%) and supporting the low frequency of acute HAV infection across age strata. No positive cases detected among infants younger than 1 year

Anti-HAV IgG seropositivity varied significantly across age groups (*p* < 0.001) (Table 1; Fig. 1). The lowest rate was observed in the 21–25-year group (31.8%). Seropositivity then increased steadily with age, surpassing 70% in the 36–40-year group and reaching > 88% among individuals aged ≥ 41 years. This pattern indicates a distinct breakpoint in early adulthood, with lower immunity in younger cohorts and high seroprevalence in older age groups.

**Table 1:** Age-specific distribution of hepatitis A serology (anti-HAV IgG and IgM), 2014–2024

Ages (years)	IgG positive *n* (%), 19,357 (68.97)	IgM positive *n* (%), 72 (0.26)	Acute hepatitis A *n* (%), 19 (0.07)	Total *n* 28,067
≤ 1	150 (70.75)	0 (0)	0 (0)	212
1–5	977 (70.39)	1 (0.07)	0 (0)	1,388
6–10	934 (61.29)	10 (0.66)	4 (0.26)	1,524
11–15	643 (40.77)	4 (0.25)	2 (0.13)	1,577
16–20	675 (35.41)	3 (0.16)	1 (0.05)	1,906
21–25	1,078 (31.82)	9 (0.39)	4 (0.17)	2,310
26–30	1,082 (45.37)	6 (0.25)	3 (0.13)	2,385
31–35	1,115 (57.56)	7 (0.36)	2 (0.10)	1,937
36–40	1,431 (74.03)	1 (0.05)	1 (0.05)	1,933
41–50	3,174 (88.04)	10 (0.28)	2 (0.06)	3,605
51–60	3,352 (97.24)	9 (0.26)	0 (0)	3,447
≥ 60	4,746 (99.66)	12 (0.25)	0 (0)	4,763
*P* values	< 0.001	0.094	0.036	–

*P* values were calculated using chi-square test for proportions across age groups.

Anti-HAV IgM positivity showed no consistent age-dependent trend. The highest rate was observed in the 6–10-year group (0.66%), while no IgM positivity was detected in infants < 1 year. Differences across age groups were not statistically significant (*p* = 0.094) (Table 1; Fig. 1).

Among the 19 clinically confirmed acute hepatitis A cases, the mean age was 23.6 ± 11.2 years (range 7–48), and 68.4% were male. Acute cases differed significantly across age groups (*p* = 0.036). Because of sparse counts in several strata, age groups were collapsed into broader bands (< 15, 16–30, 31–60, ≥ 60), which confirmed the association (*p* = 0.031). Most acute cases clustered in childhood and young adulthood (6–30 years). Seasonal clustering was observed, with 42% of acute cases occurring in August and September.

### Clinical classification of IgM-positive cases and cut-off index values

Of the 72 IgM-positive individuals, the remaining 53 (73.6%) were classified as asymptomatic/unlikely acute hepatitis A or possible false-positive reactivity (Table 2). Underlying conditions were identified in a subset, including HBV/HCV infection (*n* = 7, 9.7%), EBV infection (*n* = 4, 5.6%), autoimmune disease (*n* = 7, 9.7%), and malignancy (*n* = 9, 12.5%); no associated clinical condition was identified in 26 patients (36.1%).

Cut-off index values were significantly higher in clinically confirmed acute hepatitis A cases than in other IgM-reactive groups (median 12.00 (1.29–17.23) vs. others, *p* < 0.001). However, EBV-associated and clinically uncorrelated cases occasionally exhibited high cut-off index values (Table 2**).**

**Table 2:** Clinical classification of anti-HAV IgM–reactive cases and corresponding cut-off index values

Groups	*n* (%)	S/CO index, median (range)
Total	72 (100.0)	1.76 (1.21–18.82)
Confirmed acute HAV	19 (26.4)	12.00 (1.29–17.23)
HBV/HCV infection	7 (9.7)	1.93 (1.59–11.75)
EBV infection	4 (5.6)	8.25 (1.66–11.30)
Autoimmune disease	7 (9.7)	1.45 (1.21–1.65)
Malignancy	9 (12.5)	1.61 (1.29–6.01)
Clinically incompatible	26 (36.1)	7.42 (1.21–18.82)

Note: Percentages are calculated among anti-HAV IgM–reactive individuals (*n* = 72). Acute hepatitis A was confirmed in 19 patients (26.4%) based on anti-HAV IgM reactivity together with a clinically compatible presentation and supportive biochemical evidence (elevated ALT and/or total bilirubin). The remaining IgM-reactive cases were classified as asymptomatic/unlikely acute hepatitis A or possible false-positive reactivity, frequently in the context of other infections or underlying diseases

*S/CO:* signal-to-cutoff

### Yearly distribution and pediatric trends

**Fig. 2:**
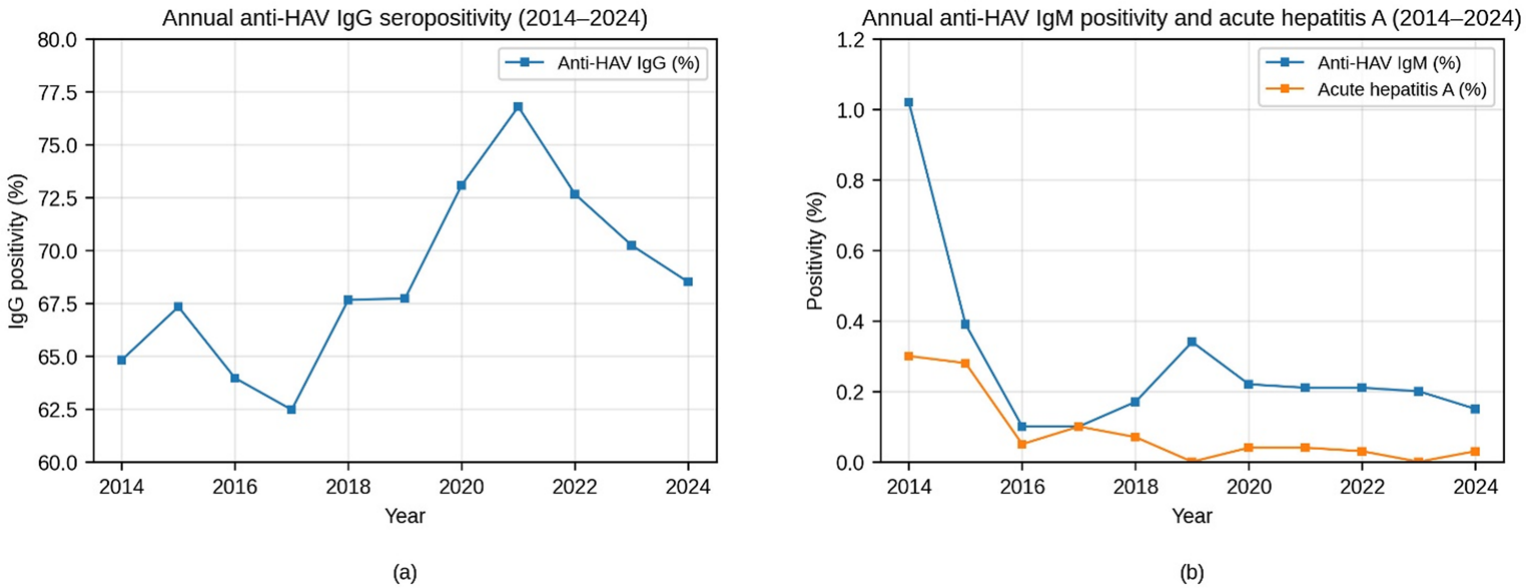
Annual distribution of anti-HAV IgG seropositivity and anti-HAV IgM positivity, with the proportion of clinically confirmed acute hepatitis A cases, 2014–2024

Anti-HAV IgG seropositivity differed significantly by year (*p* < 0.001), ranging from 62.46% to 76.79%, with the highest annual seropositivity observed in 2021 (76.79%) (Table 3). Anti-HAV IgM positivity also varied significantly over time (*p* < 0.001), peaking in 2014 (1.02%) and remaining ≤ 0.39% thereafter, with annual rates between 0.10% and 0.34% during 2016–2024 (Fig. 2). Clinically confirmed acute hepatitis A was most frequent in 2014 (0.30%) and 2015 (0.28%), but remained very low in subsequent years (0.00%–0.10%) (*p* < 0.001). Although the number of patients tested generally increased over time, testing volume declined in 2020–2021 (COVID-19 period), and IgG seropositivity peaked in 2021 (76.79%).

**Table 3:** Annual distribution of anti-HAV IgG and anti-HAV IgM results (2014–2024)

Year	Total tested *n*	IgG positive *n* (%)	IgM positive *n* (%)	Acute hepatitis A *n* (%)
Total	28,067	19,357 (68.97)	72 (0.26)	19 (0.07)
2014	1,668	1,081 (64.81)	17 (1.02)	5 (0.30)
2015	1,815	1,222 (67.33)	7 (0.39)	5 (0.28)
2016	2,006	1,283 (63.96)	2 (0.10)	1 (0.05)
2017	2,083	1,301 (62.46)	2 (0.10)	2 (0.10)
2018	2,947	1,994 (67.66)	5 (0.17)	2 (0.07)
2019	3,234	2,190 (67.72)	11 (0.34)	0 (0.00)
2020	2,303	1,683 (73.08)	5 (0.22)	1 (0.04)
2021	2,344	1,800 (76.79)	5 (0.21)	1 (0.04)
2022	2,926	2,126 (72.66)	6 (0.21)	1 (0.03)
2023	3,427	2,407 (70.24)	7 (0.20)	0 (0.00)
2024	3,314	2,270 (68.50)	5 (0.15)	1 (0.03)

**Fig. 3:**
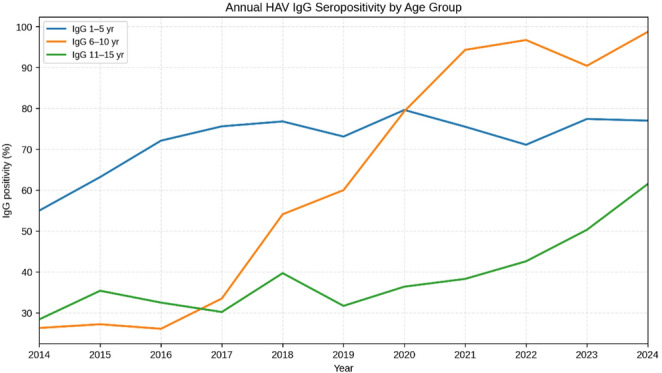
Temporal trends in anti-HAV IgG seropositivity in pediatric age groups (1–5, 6–10, and 11–15 years), 2014–2024. Anti-HAV IgG seropositivity increased steadily across all pediatric age groups over the study period. A pronounced rise was observed in the 6–10-year group from 2018 onward, consistent with the progression of vaccine-eligible birth cohorts into this age range. The 1–5-year group showed a gradual upward trend, reflecting continuous inclusion of newly vaccinated children. In the 11–15-year group, seropositivity increased more noticeably after 2021 as vaccinated cohorts aged into adolescence

Among children aged 1–15 years, annual age-stratified analysis showed a progressive increase in IgG seropositivity across pediatric age groups over the study period, with the most pronounced rise in the 6–10-year cohort (from 26.3% to 98.7%) (Table 4; Fig. 3). In contrast, IgM positivity remained extremely low in children: only 14 IgM-positive cases occurred among those aged 1–15 years across 2014–2024, and annual pediatric IgM positivity ranged from 0 to 3.5% depending on the age-year stratum. When children born in 2012 or later (eligible for the national routine vaccination program) were considered vaccine-eligible, anti-HAV IgG seropositivity in this cohort was 93.3%, and no anti-HAV IgM positivity was detected.

**Table 4:** Annual, age-stratified anti-HAV IgG seropositivity and anti-HAV IgM positivity in children aged 1–15 years (2014–2024)

Years	IgG positive (%)				IgM positive (%*)		
	1–5	6–10	11–15		1–5	6–10	11–15
2014	55.0	26.3	28.4		0.62	3.54	0.00
2015	63.2	27.2	35.4		0.00	0.49	0.56
2016	72.1	26.1	32.5		0.00	0.53	0.00
2017	75.6	33.5	30.2		0.00	0.00	0.00
2018	76.8	54.1	39.7		0.00	0.00	0.00
2019	73.1	60.0	31.7		0.00	0.00	0.00
2020	79.6	79.3	36.4		0.00	0.00	1.01
2021	75.5	94.3	38.3		0.00	0.00	0.00
2022	71.1	96.7	42.6		0.00	0.00	1.42
2023	77.4	90.4	50.3		0.00	0.00	0.00
2024	77.0	98.7	61.5		0.00	0.00	0.00

* Anti-HAV IgM positivity was calculated using the total number of children tested in the corresponding age group and year (denominator: all tested children in that stratum). In the overall cohort (*n* = 28,067), 72 individuals were anti-HAV IgM–positive, of whom 14 were children aged 1–15 years

## Discussion

In recent years, the global incidence of HAV infection has declined substantially, driven mainly by improvements in hygiene and sanitation and by the expansion of vaccination programs [5, 6]. In this study, we assessed HAV seroprevalence, its distribution across age groups and calendar years, and the impact of the national childhood vaccination program in Bursa (northwestern Türkiye) using tertiary-care hospital data collected over an 11-year period. The key findings were an overall anti-HAV IgG seroprevalence of 68.97% (consistent with intermediate endemicity), a marked susceptibility gap among young adults (31.8% in the 21–25-year group), high seropositivity with no IgM-positive cases in vaccine-eligible children, and a very low prevalence of clinically confirmed acute hepatitis A (0.07%). Overall, our findings support the effectiveness of routine childhood immunisation while highlighting the need to address residual susceptibility in specific young adult cohorts. Such seroepidemiological data are essential for identifying risk groups and informing vaccination strategies.

In Türkiye, the frequency of HAV infections is lower in the western and central regions and higher in the eastern and southern regions, indicating significant geographic heterogeneity [8]. According to province-based data, seroprevalence was 67.23% in Istanbul (2020–2023; *n* = 33,683), while a study from an eastern province (Erzurum) reported a higher seroprevalence (87.3%; *n* = 25,007), demonstrating regional variability within the country [13, 14]. The overall HAV seroprevalence observed in Bursa (68.97%) closely approximates the most recent estimate from Istanbul, supporting Bursa’s classification as an intermediate-endemic setting [13]. However, regional averages may obscure age-specific susceptibility patterns that have important public health implications.

When stratified by age, seroprevalence was relatively high in early childhood but decreased significantly in young adults. The lowest seroprevalence was observed in the 21–25-year group (31.82%), while the highest was seen in individuals aged ≥ 60 years (99.66%). This distinct breakpoint in early adulthood likely reflects a transition in which childhood exposure has decreased while older cohorts retain high cumulative immunity. This is important because symptomatic disease and the risk of severe outcomes increase with age [2, 3, 6]. The vulnerability of young adults in our cohort can plausibly be explained by their ineligibility for the national routine program introduced in 2012, leaving a cohort that was neither naturally immunized through frequent childhood exposure nor protected by routine vaccination. Similar age-patterns have been reported in Türkiye, with consistently high seroprevalence in older adults and evidence of changing susceptibility in younger groups [13–15]. For example, the Istanbul study reported seroprevalence rates above 94% after age 45 and 98.3% among those ≥ 60 years, while vaccination increased seroprevalence to 91.8% among 2–5-year-olds [13]. Similarly, a study from Erzurum, an eastern province of Türkiye, reported high seropositivity across age groups, with rates reaching 99.3% among older adults [14]. Collectively, these findings indicate that young adults remain a key susceptible group in Türkiye despite high overall seroprevalence in older populations.

In our study, anti-HAV IgG positivity was slightly higher among males than females (70.5% vs. 67.4%, *p* < 0.001). Although statistically significant, the clinical relevance of this difference appears limited, and prior studies have reported inconsistent sex-based patterns [3–15].

The number of patients tested for HAV serology increased over time, but a marked decline occurred in 2020, coinciding with the COVID-19 pandemic. This decrease is consistent with reduced hospital admissions during the pandemic [16]. Overall, HAV seroprevalence showed a significant upward trend across the 11-year period (*p* < 0.001), likely reflecting both cumulative immunity in older cohorts and the implementation of routine childhood vaccination in 2012 [9, 15]. The apparent increase in 2020–2021 may, at least in part, reflect changes in health-care–seeking behavior and testing practices (i.e., fewer tests with a higher proportion of clinically suspected cases). The subsequent decline after 2022 may be related to normalization of hospital utilization and testing denominators; additionally, disruptions in routine immunization services during the pandemic and vaccine hesitancy may have resulted in missed childhood vaccinations [17].

Among individuals born in 2012 or later (vaccine-eligible cohort), seroprevalence reached 93.3%, and no anti-HAV IgM positivity was detected. Although individual vaccination records were unavailable, this high seropositivity strongly suggests the effectiveness of routine childhood immunization. The fact that seropositivity did not reach 100% may reflect incomplete vaccination coverage or vaccine refusal, consistent with reports indicating variable hepatitis A vaccine uptake [12, 17]. A large-scale study from Argentina similarly demonstrated higher seroprevalence among children born after the introduction of the vaccination program (77.7% vs. 56.7%), with all IgM-positive cases restricted to the unvaccinated cohort [18]. Consistent with these observations, post-vaccination data from Türkiye also suggest an age-shift in susceptibility toward adolescents, supporting the population-level impact of routine childhood immunization [15].

Even in transitioning or low-incidence settings, outbreaks may re-emerge when immunity is uneven, particularly among mobile or vulnerable populations [19, 20]. For example, in Greece, 1,193 hepatitis A cases were reported during 2009–2018, including substantial contributions from migrants/refugees (*n* = 320), Roma communities (*n* = 240), and travellers (*n* = 112), illustrating how heterogeneous immunity can sustain outbreaks in Europe [21]. In Türkiye, HAV seroepidemiology remains closely linked to sanitation, socioeconomic conditions, and vaccination policies; nevertheless, evidence indicates a convergent transition characterized by reduced childhood exposure and an expanding susceptibility gap in adolescents and young adults [6, 8, 15]. Similar susceptibility pockets have been described in neighbouring or comparable settings. In Iran, anti-HAV IgG seropositivity reached 94% among chronic HBV patients (2016–2017), yet seronegativity clustered in younger adults (< 35 years), indicating persistent immunity gaps in vaccine-ineligible cohorts [22]. In Jordan, overall HAV seroprevalence was 38.3%, while seropositivity remained low in children (12.3% at ≤ 15 years), consistent with later-age susceptibility in transitioning endemicity settings [23]. Moreover, European/Mediterranean reports illustrate that even in low-incidence contexts, outbreaks can re-emerge when immunity is uneven, particularly in mobile or vulnerable populations [19–21]. These observations reinforce the need to identify and monitor susceptible cohorts beyond childhood vaccination targets.

The overall anti-HAV IgM positivity rate in our study was 0.26% (72/28,067), similar to another report from Türkiye (0.2%) [13]. However, only 19 (26.4%) IgM-positive patients were classified as acute hepatitis A based on compatible clinical and laboratory findings, corresponding to a prevalence of 0.07% in the overall study population. Acute cases were more common in the 6–10 and 21–25-year age groups, indicating exposure risk during school age and early adulthood. No acute cases were detected after age 46, likely due to very high IgG seroprevalence in older groups. Other Turkish studies have likewise reported the highest burden of acute hepatitis A among adolescents and young adults [13, 14], while in lower-income settings early childhood remains the most affected age group [24], likely reflecting early exposure due to poor sanitation and unsafe water.

Apparent false-positive anti-HAV IgM reactivity was also observed, consistent with previous reports. Among IgM-reactive patients without a compatible acute hepatitis A presentation, several had underlying conditions such as HBV/HCV infection, EBV infection, autoimmune diseases, or malignancies. In patients with these conditions, assay cross-reactivity and nonspecific IgM responses have been reported [25, 26], highlighting the importance of interpreting anti-HAV IgM results in conjunction with clinical findings and supportive liver biochemistry and, when available, molecular testing.

## Strengths and limitations

The strengths of this study include its large sample size and 11-year observation period, allowing robust age-specific and temporal analyses. In addition, the availability of both anti-HAV IgG and anti-HAV IgM results enabled simultaneous assessment of population immunity and acute infection.

Several limitations should also be acknowledged. The retrospective design may have introduced information bias and limited control over potential confounders. Individual vaccination records were unavailable, precluding confirmation of vaccination status at the patient level and limiting causal inference regarding vaccine impact. The study was conducted at a single tertiary-care university hospital; although it serves as a major referral center, the findings may not fully represent the general community. Finally, HAV serology in our institution is frequently requested not only for symptomatic patients but also for immunity screening, particularly in children. Because the clinical indication for testing could not be retrieved, the study population likely included a substantial proportion of individuals without acute hepatitis symptoms, which may have influenced estimates of acute infection, thereby reducing, though not eliminating, the possibility of missed asymptomatic infections.

## Conclusion

In conclusion, with an overall HAV seroprevalence of 68.97%, Bursa remains in the intermediate endemicity range, consistent with national patterns. High IgG seropositivity (93.3%) and the absence of anti-HAV IgM positivity among individuals born in 2012 or later (vaccine-eligible cohort) strongly suggest sustained effectiveness of the national childhood vaccination program. At the same time, very low IgM positivity (0.26%) and a low prevalence of clinically confirmed acute hepatitis A (0.07%) indicate that acute infection has become uncommon in this setting, although sporadic cases continue to occur. The presence of false-positive IgM results further underscores the importance of interpreting serology alongside clinical findings and supportive biochemical and, where available, molecular, evidence.

Overall, this study provides an updated decade-long overview of HAV seroepidemiology in Bursa. Ongoing serosurveillance and incorporation of age-specific seroprevalence data into immunization strategies will be important to sustain long-term control of hepatitis A and to inform public health policies in Türkiye.

## Data Availability

The data are not publicly available due to patient confidentiality but are available upon request to the corresponding author in de-identifiable form.
